# 5-Hy­droxy-2-nitro­benzaldehyde thio­semicarbazone (HNBATSC)

**DOI:** 10.1107/S1600536814015098

**Published:** 2014-07-02

**Authors:** M. Sivasankar Reddy, Y. Sarala, M. Jagadeesh, Samar K. Das, Varada Reddy Ammireddy

**Affiliations:** a Department of Chemistry, Sri Venkateswara University, Tirupati 517 502, India; b Department of Chemistry, Chaitanya Bharathi Institute of Technology, Gandipet, Hyderabad 500 075, India; c School of Chemistry, University of Hyderabad, Hyderabad 500 046, India

**Keywords:** Thio­semicarbazone, Evolution of mol­ecular structure, Crystallographic studies, Triclinic symmetry, crystal structure

## Abstract

The asymmetric unit of the title compound, C_8_H_8_N_4_O_3_S, consists of two independent mol­ecules. Each mol­ecule is approximately planar with dihedral angles of 8.71 (3) and 1.50 (2)° between the aromatic ring and the thio­semicarbazide moiety while the NO_2_ group makes dihedral angles of 29.27 (3) and 17.78 (3)° with the benzene ring. In the crystal, the molecules are linked by N—H⋯S, O—H⋯O and N—H⋯O hydrogen bonds, forming two-dimensional networks parallel to (100).

## Related literature   

For the crystal structures of similar Schiff base compounds see: Chattopadhyay *et al.* (1988 ▶). For the structure of 2-hy­droxy-5-nitro­benzaldehyde thio­semicarbazone, see: Alhadi *et al.* (2008 ▶). For general background to the biological activity and anti-tumour activity of benzaldehyde thiosemicarbazone derivatives, see: Hamre *et al.* (1950 ▶); Brockman *et al.* (1956 ▶).
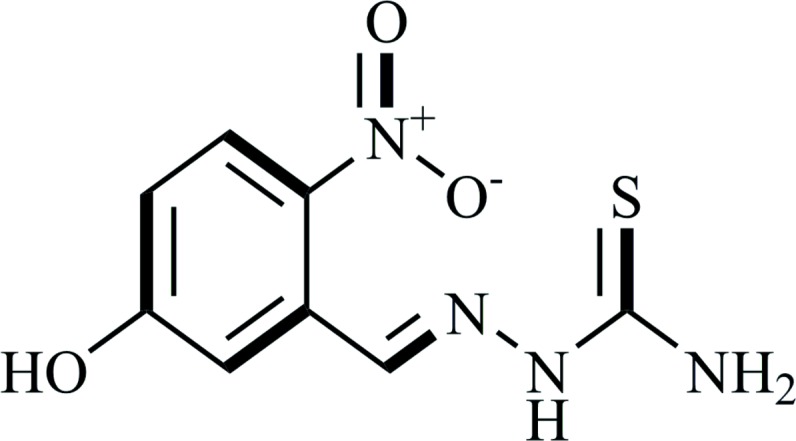


## Experimental   

### 

#### Crystal data  

C_8_H_8_N_4_O_3_S
*M* *_r_* = 240.24
Triclinic, 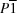

*a* = 7.1328 (13) Å
*b* = 8.0738 (15) Å
*c* = 17.868 (3) Å
α = 102.142 (16)°
β = 94.325 (15)°
γ = 95.212 (15)°
*V* = 997.1 (3) Å^3^
*Z* = 4
Mo *K*α radiation
μ = 0.32 mm^−1^
*T* = 298 K
0.30 × 0.20 × 0.14 mm

#### Data collection  

Agilent Xcalibur (Eos, Gemini) diffractometer
Absorption correction: multi-scan (*CrysAlis PRO*; Agilent, 2013 ▶) *T*_min_ = 0.796, *T*_max_ = 1.000
7083 measured reflections
4063 independent reflections
1973 reflections with *I* > 2σ(*I*)
*R*_int_ = 0.063

#### Refinement  

*R*[*F* ^2^ > 2σ(*F* ^2^)] = 0.065
*wR*(*F* ^2^) = 0.137
*S* = 0.99
4063 reflections
321 parameters
H atoms treated by a mixture of independent and constrained refinement
Δρ_max_ = 0.28 e Å^−3^
Δρ_min_ = −0.33 e Å^−3^

### 

Data collection: *CrysAlis PRO* (Agilent, 2013 ▶); cell refinement: *CrysAlis PRO*; data reduction: *CrysAlis PRO*; program(s) used to solve structure: *SHELXS97* (Sheldrick, 2008 ▶); program(s) used to refine structure: *SHELXL97* (Sheldrick, 2008 ▶); molecular graphics: *SHELXTL* (Sheldrick, 2008 ▶); software used to prepare material for publication: *publCIF* (Westrip, 2010 ▶).

## Supplementary Material

Crystal structure: contains datablock(s) I. DOI: 10.1107/S1600536814015098/ds2241sup1.cif

Structure factors: contains datablock(s) I. DOI: 10.1107/S1600536814015098/ds2241Isup2.hkl

Supporting information file. DOI: 10.1107/S1600536814015098/ds2241Isup3.cml

CCDC reference: 1010403

Additional supporting information:  crystallographic information; 3D view; checkCIF report

## Figures and Tables

**Table 1:** Hydrogen-bond geometry (Å, °)

*D*—H⋯*A*	*D*—H	H⋯*A*	*D*⋯*A*	*D*—H⋯*A*
N8—H8*B*⋯S1^i^	0.80 (6)	2.59 (6)	3.323 (5)	153 (6)
N7—H7*N*⋯S1^ii^	1.07 (4)	2.22 (4)	3.264 (4)	162 (3)
N3—H3*N*⋯S2^iii^	0.95 (4)	2.41 (4)	3.324 (4)	160 (4)
N4—H4*B*⋯S2^iv^	0.86 (4)	2.51 (4)	3.373 (4)	180 (4)
O4—H4*O*⋯O5^iii^	0.76 (5)	2.27 (6)	2.960 (5)	153 (7)
C3—H3⋯O9^v^	0.93	2.57	3.491 (6)	168
C7—H7⋯O5^v^	0.93	2.79	3.295 (5)	115
N4—H4*A*⋯O7^iii^	0.87 (4)	2.48 (4)	3.066 (5)	125 (3)
O6—H6*O*⋯O4^vi^	1.01 (5)	1.81 (5)	2.810 (5)	170 (4)
N8—H8*A*⋯O9^ii^	0.98 (5)	2.39 (4)	3.002 (5)	120 (3)
C13—H13⋯O10^ii^	0.93	2.67	3.506 (5)	150

Symmetry codes: (i) 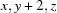; (ii) 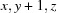; (iii) 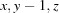; (iv) 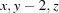; (v) 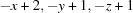; (vi) 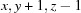.

**Fig. 1.**
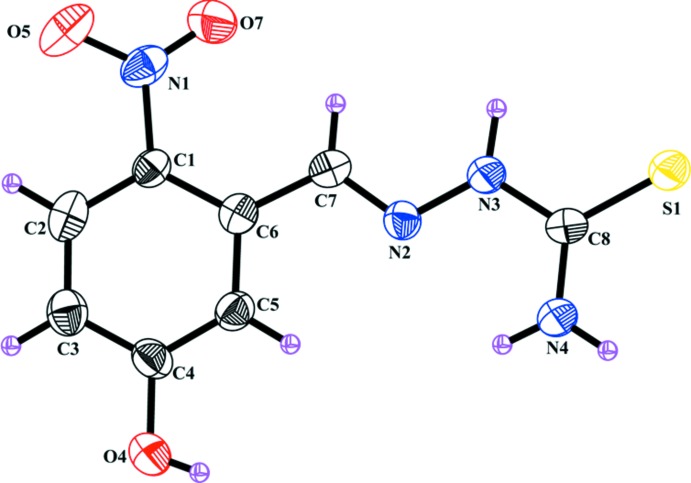
ORTEP view of one of the independent molecules of the title compound. Thermal ellipsoids are at the 50% probability level.

**Fig. 2.**
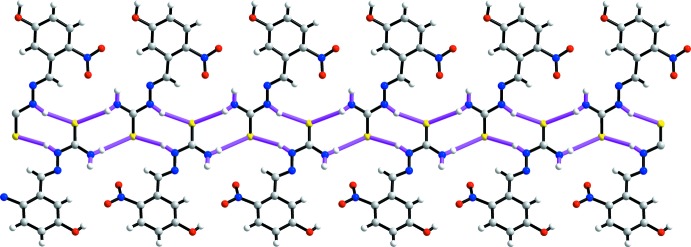
one-dimensional chain formed due to N—H···S interactions.

**Fig. 3.**
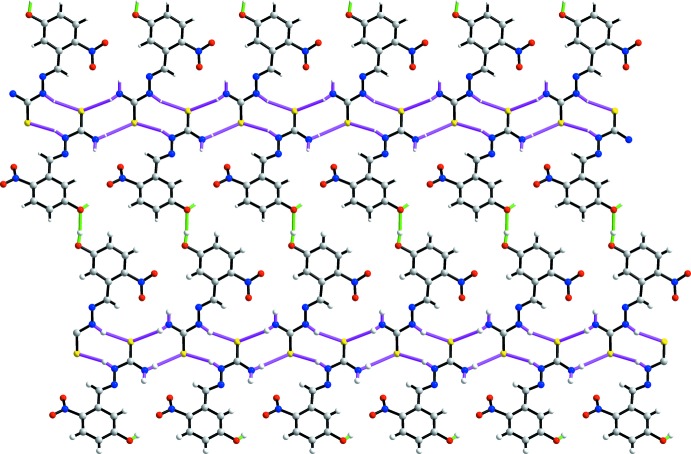
Combination of O—H···O and N—H···S interactions leading to the formation of two-dimensional corrugated sheet.

## supplementary crystallographic information

### S1. Comment

Benzaldehyde­thio­semicarbazone derivatives show *in vitro* anti-bacterial, anti-oxidant and anti-tubercular activities. Thio­semicarbazones have also been used as second line drugs in the chemotherapy of leprosy. Since then, several workers have reported the anti-microbial activity of thio­semicarbazones against selected plant pathogenic and saprophytic fungi. The anti-viral effect of thio­semicarbazones was first demonstrated (Hamre *et al.*, 1950). They explained that p-amino­benzaldehyde-3-thio­semicarbazone and several of its derivatives were active against vaccinia virus in mice. Anti­tumor activity against leukemia in mice was first reported (Brockman *et al.*, 1956).

We reported here the synthesis and structural characterization of a Schiff base, 5-hy­droxy-2-nitro­benzaldehyde­thio­semicarbazone (Fig. 1). Due to the presence of potential hydrogen donor sites in the molecule, supra­molecular hydrogen bonding inter­actions in the domain of thio­semicarbazones are observed. Inter­molecular N—H···S inter­actions through R^2^_2_(8) synthons result in the formation of 1D chains (Fig. 2). These 1D chains, with the aid of O—H···O inter­actions, form 2D corrugated sheets (Fig.3).

### S2. Experimental

5-Hy­droxy-2-nitro­benzaldehyde­thio­semicarbazone (0.33 g, 2 mmol) and thio­semicarbazide (0.18 g, 2 mmol) were separately dissolved in 20 ml of ethanol and subsequently they were mixed. The resulting mixture (40 ml) was refluxed for 5 hrs. The precipitate, formed during this time, was filtered and washed with a small amount of ethanol. The purity of the product HNBATSC was checked by TLC. Finally HNBATSC was dissolved in aceto­nitrile and then slowly it was evaporated for the removal of aceto­nitrile to obtain white crystals.

### S2.1. Refinement

Crystal data, data collection and structure refinement details are summarized in Table 1.

### Crystal data

**Table:**

C_8_H_8_N_4_O_3_S	*Z* = 4
*M**_r_* = 240.24	*F*(000) = 496
Triclinic, *P*1	*D*_x_ = 1.600 Mg m^−^^3^
Hall symbol: -P 1	Melting point: 538 K
*a* = 7.1328 (13) Å	Mo *K*α radiation, λ = 0.71073 Å
*b* = 8.0738 (15) Å	Cell parameters from 858 reflections
*c* = 17.868 (3) Å	θ = 2.9–23.0°
α = 102.142 (16)°	µ = 0.32 mm^−^^1^
β = 94.325 (15)°	*T* = 298 K
γ = 95.212 (15)°	Block, colorless
*V* = 997.1 (3) Å^3^	0.30 × 0.20 × 0.14 mm

### Data collection

**Table:**

Agilent Xcalibur (Eos, Gemini) diffractometer	1973 reflections with *I* > 2σ(*I*)
Radiation source: fine-focus sealed tube	*R*_int_ = 0.063
Graphite monochromator	θ_max_ = 26.4°, θ_min_ = 2.9°
ω scans	*h* = −8→5
Absorption correction: multi-scan (*CrysAlis PRO*; Agilent, 2013)	*k* = −10→9
*T*_min_ = 0.796, *T*_max_ = 1.000	*l* = −22→21
7083 measured reflections	3904 standard reflections every 0 reflections
4063 independent reflections	intensity decay: none

### Refinement

**Table:**

Refinement on *F*^2^	Primary atom site location: structure-invariant direct methods
Least-squares matrix: full	Secondary atom site location: difference Fourier map
*R*[*F*^2^ > 2σ(*F*^2^)] = 0.065	Hydrogen site location: inferred from neighbouring sites
*wR*(*F*^2^) = 0.137	H atoms treated by a mixture of independent and constrained refinement
*S* = 0.99	*w* = 1/[σ^2^(*F*_o_^2^) + (0.0237*P*)^2^] where *P* = (*F*_o_^2^ + 2*F*_c_^2^)/3
4063 reflections	(Δ/σ)_max_ < 0.001
321 parameters	Δρ_max_ = 0.28 e Å^−^^3^
0 restraints	Δρ_min_ = −0.33 e Å^−^^3^

### Special details

**Table:**

Experimental. Absorption correction: (CrysAlisPro, Agilent Technologies, 2013) Version 1.171.36.28 (release 01-02-2013 CrysAlis171 .NET) (compiled Feb 1 2013,16:14:44) Empirical absorption correction using spherical harmonics, implemented in SCALE3 ABSPACK scaling algorithm.
Geometry. All e.s.d.'s (except the e.s.d. in the dihedral angle between two l.s. planes) are estimated using the full covariance matrix. The cell e.s.d.'s are taken into account individually in the estimation of e.s.d.'s in distances, angles and torsion angles; correlations between e.s.d.'s in cell parameters are only used when they are defined by crystal symmetry. An approximate (isotropic) treatment of cell e.s.d.'s is used for estimating e.s.d.'s involving l.s. planes.
Refinement. Refinement of *F*^2^ against ALL reflections. The weighted *R*-factor *wR* and goodness of fit *S* are based on *F*^2^, conventional *R*-factors *R* are based on *F*, with *F* set to zero for negative *F*^2^. The threshold expression of *F*^2^ > σ(*F*^2^) is used only for calculating *R*-factors(gt) *etc*. and is not relevant to the choice of reflections for refinement. *R*-factors based on *F*^2^ are statistically about twice as large as those based on *F*, and *R*- factors based on ALL data will be even larger.

### Fractional atomic coordinates and isotropic or equivalent isotropic displacement parameters (Å^2^)

**Table:**

	*x*	*y*	*z*	*U*_iso_*/*U*_eq_	
S1	0.47754 (18)	−0.25982 (13)	0.26463 (6)	0.0460 (3)	
S2	0.46264 (19)	1.26534 (13)	0.31459 (7)	0.0502 (4)	
N3	0.6268 (5)	−0.0428 (4)	0.3896 (2)	0.0412 (10)	
N2	0.7076 (5)	−0.0018 (4)	0.46366 (19)	0.0392 (9)	
N6	0.6606 (5)	1.0485 (4)	0.1259 (2)	0.0437 (9)	
N8	0.6172 (7)	1.3598 (5)	0.1978 (3)	0.0520 (12)	
N4	0.6096 (6)	−0.3229 (5)	0.3963 (3)	0.0483 (11)	
C7	0.7530 (6)	0.1563 (5)	0.4924 (2)	0.0413 (11)	
H7	0.7302	0.2376	0.4639	0.050*	
N7	0.5833 (5)	1.0750 (4)	0.1944 (2)	0.0451 (10)	
C1	0.9021 (6)	0.3740 (5)	0.6117 (2)	0.0381 (11)	
C6	0.8416 (6)	0.2067 (5)	0.5711 (2)	0.0388 (11)	
C8	0.5768 (6)	−0.2103 (5)	0.3549 (2)	0.0363 (10)	
C16	0.5603 (6)	1.2346 (5)	0.2306 (2)	0.0405 (11)	
O4	0.9804 (6)	−0.0116 (5)	0.7225 (2)	0.0636 (12)	
C5	0.8744 (6)	0.0787 (5)	0.6102 (2)	0.0426 (11)	
H5	0.8391	−0.0344	0.5853	0.051*	
C9	0.7661 (6)	0.6841 (5)	−0.0180 (2)	0.0405 (11)	
N1	0.8718 (6)	0.5229 (5)	0.5792 (2)	0.0501 (11)	
O5	0.9645 (5)	0.6588 (4)	0.6107 (2)	0.0773 (12)	
C13	0.7647 (6)	0.9729 (5)	−0.0234 (2)	0.0428 (11)	
H13	0.7424	1.0838	−0.0020	0.051*	
C4	0.9566 (7)	0.1144 (6)	0.6837 (2)	0.0455 (12)	
O6	0.8570 (5)	1.0706 (4)	−0.1298 (2)	0.0665 (11)	
C14	0.7334 (6)	0.8488 (5)	0.0184 (2)	0.0397 (11)	
C2	0.9894 (6)	0.4091 (6)	0.6850 (3)	0.0505 (12)	
H2	1.0305	0.5213	0.7096	0.061*	
C10	0.8258 (7)	0.6471 (6)	−0.0898 (3)	0.0521 (13)	
H10	0.8436	0.5355	−0.1123	0.063*	
O7	0.7578 (6)	0.5092 (4)	0.5244 (2)	0.0791 (13)	
C12	0.8277 (7)	0.9384 (6)	−0.0954 (3)	0.0484 (12)	
C11	0.8594 (7)	0.7740 (6)	−0.1289 (3)	0.0533 (13)	
H11	0.9031	0.7499	−0.1773	0.064*	
C15	0.6601 (7)	0.8923 (5)	0.0926 (2)	0.0474 (12)	
H15	0.6129	0.8067	0.1159	0.057*	
C3	1.0168 (6)	0.2796 (6)	0.7225 (3)	0.0500 (12)	
H3	1.0744	0.3027	0.7726	0.060*	
O10	0.7007 (7)	0.4005 (4)	−0.0180 (2)	0.0960 (15)	
N5	0.7434 (6)	0.5429 (5)	0.0211 (3)	0.0558 (12)	
O9	0.7659 (6)	0.5702 (4)	0.0907 (2)	0.0748 (12)	
H4B	0.572 (6)	−0.428 (5)	0.376 (2)	0.054 (15)*	
H3N	0.598 (6)	0.035 (5)	0.358 (2)	0.069 (15)*	
H7N	0.534 (6)	0.983 (5)	0.226 (2)	0.081 (15)*	
H8A	0.667 (7)	1.338 (6)	0.147 (3)	0.080 (18)*	
H4A	0.659 (6)	−0.292 (5)	0.444 (2)	0.048 (15)*	
H8B	0.617 (9)	1.453 (7)	0.224 (4)	0.13 (3)*	
H6O	0.891 (7)	1.030 (6)	−0.184 (3)	0.082 (18)*	
H4O	0.939 (9)	−0.096 (7)	0.697 (3)	0.10 (3)*	

### Atomic displacement parameters (Å^2^)

**Table:**

	*U*^11^	*U*^22^	*U*^33^	*U*^12^	*U*^13^	*U*^23^
S1	0.0546 (9)	0.0391 (6)	0.0446 (8)	0.0091 (6)	0.0054 (6)	0.0076 (5)
S2	0.0669 (10)	0.0391 (6)	0.0465 (8)	0.0115 (6)	0.0140 (7)	0.0084 (5)
N3	0.050 (3)	0.035 (2)	0.039 (2)	0.0082 (19)	0.0018 (19)	0.0072 (18)
N2	0.042 (2)	0.039 (2)	0.036 (2)	0.0052 (18)	0.0036 (18)	0.0061 (17)
N6	0.048 (3)	0.043 (2)	0.040 (2)	0.0083 (19)	0.0091 (19)	0.0047 (18)
N8	0.074 (3)	0.034 (2)	0.048 (3)	0.004 (2)	0.016 (2)	0.006 (2)
N4	0.064 (3)	0.034 (2)	0.046 (3)	0.010 (2)	−0.002 (2)	0.007 (2)
C7	0.043 (3)	0.037 (2)	0.047 (3)	0.005 (2)	0.008 (2)	0.012 (2)
N7	0.057 (3)	0.0358 (19)	0.045 (2)	0.007 (2)	0.014 (2)	0.0111 (19)
C1	0.037 (3)	0.034 (2)	0.042 (3)	0.002 (2)	0.008 (2)	0.007 (2)
C6	0.032 (3)	0.041 (2)	0.043 (3)	0.003 (2)	0.007 (2)	0.008 (2)
C8	0.030 (3)	0.038 (2)	0.045 (3)	0.007 (2)	0.009 (2)	0.015 (2)
C16	0.047 (3)	0.038 (2)	0.038 (3)	0.005 (2)	0.001 (2)	0.012 (2)
O4	0.092 (3)	0.053 (2)	0.043 (2)	0.009 (2)	−0.010 (2)	0.0098 (19)
C5	0.048 (3)	0.035 (2)	0.044 (3)	0.005 (2)	0.006 (2)	0.006 (2)
C9	0.044 (3)	0.040 (2)	0.039 (3)	0.008 (2)	0.004 (2)	0.010 (2)
N1	0.062 (3)	0.036 (2)	0.054 (3)	0.009 (2)	0.024 (2)	0.008 (2)
O5	0.079 (3)	0.0408 (19)	0.109 (3)	−0.005 (2)	0.014 (2)	0.012 (2)
C13	0.043 (3)	0.039 (2)	0.048 (3)	0.007 (2)	0.005 (2)	0.012 (2)
C4	0.053 (3)	0.051 (3)	0.034 (3)	0.009 (3)	0.004 (2)	0.011 (2)
O6	0.085 (3)	0.067 (2)	0.052 (2)	0.003 (2)	0.018 (2)	0.023 (2)
C14	0.044 (3)	0.037 (2)	0.038 (3)	0.004 (2)	0.002 (2)	0.009 (2)
C2	0.044 (3)	0.044 (3)	0.057 (3)	−0.002 (2)	0.011 (3)	−0.004 (2)
C10	0.063 (4)	0.049 (3)	0.042 (3)	0.018 (3)	0.004 (3)	0.000 (2)
O7	0.122 (4)	0.056 (2)	0.059 (3)	0.019 (2)	−0.011 (2)	0.015 (2)
C12	0.043 (3)	0.060 (3)	0.045 (3)	0.006 (3)	0.006 (2)	0.018 (3)
C11	0.059 (4)	0.063 (3)	0.041 (3)	0.018 (3)	0.016 (3)	0.008 (2)
C15	0.061 (4)	0.038 (2)	0.044 (3)	0.004 (2)	0.007 (3)	0.011 (2)
C3	0.042 (3)	0.055 (3)	0.050 (3)	0.000 (3)	0.001 (2)	0.008 (3)
O10	0.147 (4)	0.0392 (19)	0.097 (3)	0.009 (2)	0.014 (3)	0.005 (2)
N5	0.073 (3)	0.038 (2)	0.055 (3)	0.011 (2)	0.007 (2)	0.005 (2)
O9	0.113 (4)	0.063 (2)	0.056 (2)	0.020 (2)	0.019 (2)	0.023 (2)

### Geometric parameters (Å, º)

**Table:**

S1—C8	1.664 (4)	C4—C3	1.381 (6)
S2—C16	1.682 (4)	O6—C12	1.347 (5)
N3—N2	1.364 (4)	C14—C15	1.448 (6)
N3—C8	1.366 (5)	C2—C3	1.376 (5)
N2—C7	1.274 (5)	C10—C11	1.370 (5)
N6—C15	1.276 (5)	C12—C11	1.382 (6)
N6—N7	1.365 (5)	O10—N5	1.212 (4)
N8—C16	1.321 (5)	N5—O9	1.212 (4)
N4—C8	1.312 (5)	N3—H3N	0.96 (4)
C7—C6	1.457 (5)	N8—H8A	0.98 (5)
N7—C16	1.345 (5)	N8—H8B	0.80 (6)
C1—C2	1.368 (5)	N4—H4B	0.86 (4)
C1—C6	1.403 (5)	N4—H4A	0.87 (4)
C1—N1	1.467 (5)	C7—H7	0.9300
C6—C5	1.390 (5)	N7—H7N	1.07 (4)
O4—C4	1.362 (5)	O4—H4O	0.76 (5)
C5—C4	1.359 (5)	C5—H5	0.9300
C9—C10	1.363 (5)	C13—H13	0.9300
C9—C14	1.401 (5)	O6—H6O	1.00 (5)
C9—N5	1.461 (5)	C2—H2	0.9300
N1—O7	1.204 (4)	C10—H10	0.9300
N1—O5	1.229 (4)	C11—H11	0.9300
C13—C12	1.377 (5)	C15—H15	0.9300
C13—C14	1.381 (5)	C3—H3	0.9300
			
N2—N3—C8	119.2 (3)	C13—C12—C11	119.9 (4)
C7—N2—N3	116.2 (3)	C10—C11—C12	119.2 (4)
C15—N6—N7	114.8 (4)	N6—C15—C14	119.7 (4)
N2—C7—C6	118.4 (4)	C2—C3—C4	118.1 (4)
C16—N7—N6	119.7 (3)	O9—N5—O10	122.0 (4)
C2—C1—C6	122.1 (4)	O9—N5—C9	120.0 (4)
C2—C1—N1	115.5 (4)	O10—N5—C9	118.0 (4)
C6—C1—N1	122.3 (4)	N2—N3—H3N	127 (3)
C5—C6—C1	116.0 (4)	C8—N3—H3N	114 (3)
C5—C6—C7	117.9 (4)	C16—N8—H8A	122 (3)
C1—C6—C7	126.2 (4)	C16—N8—H8B	114 (4)
N4—C8—N3	116.9 (4)	H8A—N8—H8B	124 (5)
N4—C8—S1	124.0 (4)	C8—N4—H4B	117 (3)
N3—C8—S1	119.0 (3)	C8—N4—H4A	122 (3)
N8—C16—N7	117.4 (4)	H4B—N4—H4A	121 (4)
N8—C16—S2	123.4 (3)	N2—C7—H7	120.8
N7—C16—S2	119.2 (3)	C6—C7—H7	120.8
C4—C5—C6	121.7 (4)	C16—N7—H7N	112 (2)
C10—C9—C14	122.7 (4)	N6—N7—H7N	129 (2)
C10—C9—N5	116.5 (4)	C4—O4—H4O	108 (4)
C14—C9—N5	120.8 (4)	C4—C5—H5	119.1
O7—N1—O5	122.7 (4)	C6—C5—H5	119.1
O7—N1—C1	119.9 (4)	C12—C13—H13	118.8
O5—N1—C1	117.4 (4)	C14—C13—H13	118.8
C12—C13—C14	122.5 (4)	C12—O6—H6O	110 (2)
C5—C4—O4	121.2 (4)	C1—C2—H2	119.8
C5—C4—C3	121.5 (4)	C3—C2—H2	119.8
O4—C4—C3	117.3 (4)	C9—C10—H10	120.0
C13—C14—C9	115.6 (4)	C11—C10—H10	120.0
C13—C14—C15	119.6 (4)	C10—C11—H11	120.4
C9—C14—C15	124.6 (4)	C12—C11—H11	120.4
C1—C2—C3	120.5 (4)	N6—C15—H15	120.1
C9—C10—C11	120.1 (4)	C14—C15—H15	120.1
O6—C12—C13	117.0 (4)	C2—C3—H3	120.9
O6—C12—C11	123.1 (5)	C4—C3—H3	120.9
			
C8—N3—N2—C7	179.2 (4)	C10—C9—C14—C13	0.2 (7)
N3—N2—C7—C6	−179.4 (4)	N5—C9—C14—C13	178.2 (4)
C15—N6—N7—C16	−173.7 (4)	C10—C9—C14—C15	176.4 (4)
C2—C1—C6—C5	0.5 (6)	N5—C9—C14—C15	−5.7 (7)
N1—C1—C6—C5	−177.9 (4)	C6—C1—C2—C3	−1.6 (7)
C2—C1—C6—C7	−178.0 (4)	N1—C1—C2—C3	176.9 (4)
N1—C1—C6—C7	3.6 (7)	C14—C9—C10—C11	1.2 (8)
N2—C7—C6—C5	1.0 (6)	N5—C9—C10—C11	−176.8 (4)
N2—C7—C6—C1	179.4 (4)	C14—C13—C12—O6	−178.6 (4)
N2—N3—C8—N4	0.4 (6)	C14—C13—C12—C11	0.9 (7)
N2—N3—C8—S1	179.8 (3)	C9—C10—C11—C12	−1.6 (7)
N6—N7—C16—N8	−0.8 (7)	O6—C12—C11—C10	−180.0 (4)
N6—N7—C16—S2	179.3 (3)	C13—C12—C11—C10	0.6 (8)
C1—C6—C5—C4	1.3 (6)	N7—N6—C15—C14	178.1 (4)
C7—C6—C5—C4	179.9 (4)	C13—C14—C15—N6	−14.0 (7)
C2—C1—N1—O7	−161.7 (4)	C9—C14—C15—N6	170.0 (4)
C6—C1—N1—O7	16.8 (7)	C1—C2—C3—C4	1.0 (7)
C2—C1—N1—O5	17.3 (6)	C5—C4—C3—C2	0.8 (7)
C6—C1—N1—O5	−164.2 (4)	O4—C4—C3—C2	−178.3 (4)
C6—C5—C4—O4	177.1 (4)	C10—C9—N5—O9	150.5 (5)
C6—C5—C4—C3	−2.0 (7)	C14—C9—N5—O9	−27.6 (7)
C12—C13—C14—C9	−1.3 (7)	C10—C9—N5—O10	−30.0 (7)
C12—C13—C14—C15	−177.6 (4)	C14—C9—N5—O10	152.0 (5)

### Hydrogen-bond geometry (Å, º)

**Table:**

*D*—H···*A*	*D*—H	H···*A*	*D*···*A*	*D*—H···*A*
N8—H8*B*···S1^i^	0.80 (6)	2.59 (6)	3.323 (5)	153 (6)
N7—H7*N*···S1^ii^	1.07 (4)	2.22 (4)	3.264 (4)	162 (3)
N3—H3*N*···S2^iii^	0.95 (4)	2.41 (4)	3.324 (4)	160 (4)
N4—H4*B*···S2^iv^	0.86 (4)	2.51 (4)	3.373 (4)	180 (4)
O4—H4*O*···O5^iii^	0.76 (5)	2.27 (6)	2.960 (5)	153 (7)
C3—H3···O9^v^	0.93	2.57	3.491 (6)	168
C7—H7···O5^v^	0.93	2.79	3.295 (5)	115
N4—H4*A*···O7^iii^	0.87 (4)	2.48 (4)	3.066 (5)	125 (3)
O6—H6*O*···O4^vi^	1.01 (5)	1.81 (5)	2.810 (5)	170 (4)
N8—H8*A*···O9^ii^	0.98 (5)	2.39 (4)	3.002 (5)	120 (3)
C13—H13···O10^ii^	0.93	2.67	3.506 (5)	150

Symmetry codes: (i) *x*, *y*+2, *z*; (ii) *x*, *y*+1, *z*; (iii) *x*, *y*−1, *z*; (iv) *x*, *y*−2, *z*; (v) −*x*+2, −*y*+1, −*z*+1; (vi) *x*, *y*+1, *z*−1.
